# The Combined Impact on Condylar Changes of Sex and Cortication on Mandibular Condyle: A Binary Logistic Regression Analysis

**DOI:** 10.1155/ijod/5363346

**Published:** 2025-09-18

**Authors:** Agus Ertanto, Bramma Kiswanjaya, Ira Tanti, Menik Priaminiarti, Hanna H Bachtiar-Iskandar, Akihiro Yoshihara

**Affiliations:** ^1^Department of Dentomaxillofacial Radiology, Faculty of Dentistry, Universitas Indonesia, Jl Salemba Raya No. 4, Jakarta 10430, Indonesia; ^2^Department of Prosthodontics, Faculty of Dentistry, Universitas Indonesia, Jl Salemba Raya No. 4, Jakarta 10430, Indonesia; ^3^Department of Oral Health Science and Promotion, Graduate School of Medical and Dental Sciences, Niigata University, Niigata 951-8514, Japan

**Keywords:** aged, cone-beam computed tomography, sex, temporomandibular joint disorders

## Abstract

**Introduction:** Temporomandibular joint (TMJ) disorders encompass a wide range of conditions impacting the joint and its surrounding structures, often leading to pain, dysfunction, and structural alterations. Understanding the predictors of condylar changes is crucial for early diagnosis and effective management, particularly in patients experiencing significant functional limitations.

**Objectives:** This study aimed to examine the combined impact of sex and cortication classification on mandibular condylar changes, as detected by cone-beam computed tomography (CBCT), to identify key predictors for TMJ degeneration.

**Methods:** A cross-sectional study was conducted on 64 patients (33 men, 31 women) aged 30 years and older, who underwent CBCT imaging. Demographic data (age, sex), dental factors (remaining teeth count, posterior tooth loss), and anatomical measurements (condylar height, width, length, and cortication on the mandibular condyle [CMC]) were analyzed. Condylar changes were classified as osteophytes, flattening, sclerosis, erosion, or pseudocysts. Chi-square tests, independent *t*-tests, and logistic regression were applied to identify predictors of condylar changes.

**Results:** Women showed a significantly higher likelihood of condylar changes than men, with an odds ratio (OR) of 0.18, suggesting men were 82% less likely to exhibit these changes. CMC classification also played a significant role; individuals with CMC classifications of 0 or 1 had a 7.5 times higher risk of condylar changes than those with CMC class 2. Additionally, reduced condylar height was significantly associated with degenerative changes (mean height: 20.49 ± 2.5 mm in affected individuals versus 22.34 ± 3.2 mm in unaffected individuals, *p*=0.017).

**Conclusion:** The study concluded that both sex and CMC classification are significant predictors of condylar changes in TMJ disorders.

## 1. Introduction

Temporomandibular joint (TMJ) disorders encompass a variety of conditions affecting the joint and the surrounding musculature, often leading to pain, dysfunction, and structural changes, such as condylar alterations. These alterations, including osteophyte formation, flattening, sclerosis, and erosion, can be influenced by several factors, including demographic, anatomical, and dental factors [1]. Identifying predictors of these changes is therefore essential for early intervention and targeted management of TMJ disorders, as patients with severe TMJ degeneration often experience substantial limitations in mastication, speech, and other mandibular functions.

Existing research indicates that various demographic and anatomical factors may influence the onset and severity of condylar degeneration. For instance, several studies have documented a higher prevalence of TMJ disorders among women [2], suggesting that hormonal factors might contribute to these differences by modulating joint tissue integrity and pain sensitivity [3]. Anatomical characteristics, such as condylar height, width, and length [4, 5], as well as cortication on the mandibular condyle (CMC) [6], are also thought to play a role in joint health. However, despite these associations, a clear understanding of how these factors interact to influence the progression of TMJ disorders remains elusive. Furthermore, prior studies have highlighted cone-beam computed tomography (CBCT) as an effective imaging technique to observe detailed condylar morphology and pathology [7], yet the application of CBCT in the specific assessment of CMC classifications remains underexplored.

This study employs CBCT to examine the relationships between condylar changes and a variety of demographic, anatomical, and dental factors, with a particular focus on CMC classification. CBCT's high-resolution imaging enables accurate morphological assessment of condylar structure, making it possible to identify structural variations that could predict degenerative changes [7]. We hypothesize that individuals with lower CMC classifications (i.e., CMC 0 or 1) will present a higher risk of condylar alterations, as reduced cortical coverage may impair the condyle's ability to withstand functional stress, thus increasing susceptibility to degenerative changes.

To date, few studies have directly examined how the presence or absence of cortical bone on the mandibular condyle influences its susceptibility to degenerative changes. Cortication plays a key biomechanical role in distributing masticatory loads and is essential for resisting subchondral remodeling and erosion. Consequently, reduced or absent cortication may predispose the condyle to structural breakdown. Furthermore, given the known sex differences in hormonal regulation and bone remodeling, investigating the combined effects of sex and cortication on condylar morphology provides novel insight into personalized risk profiling for TMJ disorders.

The aim of this study is to examine the combined impact of sex and cortication classification on mandibular condylar changes, as detected by CBCT, to identify key predictors for TMJ degeneration. By elucidating these relationships, the study seeks to provide a more comprehensive understanding of TMJ disorder risk factors, which may support the development of sex-specific and individualized approaches in the diagnosis and management of TMJ disorders, particularly for those at high risk. This research ultimately investigates to inform clinical practice by advocating for routine assessment of CMC classification and consideration of demographic variables in TMJ evaluations to facilitate early diagnosis and preventive care for susceptible individuals.

## 2. Materials and Methods

This cross-sectional study used a dataset from CBCT CS9300 (Carestream Health Inc., Rochester, NY, USA) with 80–120 kVp, 5–11 mA, and voxel size 0.25–0.3 mm. The Digital Imaging and Communications in Medicine (DICOM) data applied had a field of view (FOV) of 17 cm × 13 cm and 17 cm × 10 cm from the Dental Radiology Clinic, Faculty of Dentistry, Universitas Indonesia. The research protocol was approved by the Dentistry Research Ethics Commission of the Faculty of Dentistry, Universitas Indonesia (Number 050981023), and was conducted in accordance with the Declaration of Helsinki. A minimum of 58 subjects was required, calculated using the cross-sectional study formula *n* = (*Zα*^2^ × *P* × *Q*)/*d*^2^, where *Zα* is a confidence interval of 95% (*α* = 0.05), *P* is a prevalence of 0.813 for condylar morphological changes derived from prior research [8], *Q* = 1 − *P*, and *d* is the absolute deviation value. To account for potential sample attrition and the statistical power needed, an additional 10% was added. Samples were obtained through consecutive sampling from patients who underwent CBCT imaging at the Dental Radiology Clinic, Faculty of Dentistry, Universitas Indonesia, from 2019 onwards. Inclusion criteria were age ≥30 years and CBCT scans with diagnostic-quality imaging of the mandibular condyle. Exclusion criteria included scans with lesions, fractures, or pathological conditions affecting the condyle or insufficient ROI visibility. A total of 69 samples were initially obtained; however, five samples were excluded on the basis of the exclusion criteria or having an incomplete ROI that could not be measured. Finally, 64 samples were obtained, consisting of 33 men and 31 women.

Intraobserver and interobserver reliability tests were conducted on 32 samples (50% of the total 64 samples). The variable condylar changes, which represents categorical data, was evaluated using the kappa agreement test, while condylar length, representing numerical data, was assessed using the intraclass correlation coefficient (ICC). These tests were performed to assess the consistency of observer ratings over time and between different observers. Intra and interobserver tests were conducted 2 weeks following the initial observations. The second observer was a lecturer of dental radiology with 17 years of experience.

### 2.1. Epidemiological and Dental Factors

The epidemiological and dental factors assessed in this study included age, sex, the number of remaining teeth, and posterior tooth loss. Age was recorded in years, and sex was categorized as men or women. The number of remaining teeth and posterior tooth loss were determined using panoramic views of CBCT radiographic imaging. For the purposes of this count, remaining teeth included those up to the third molar, whether healthy or carious, with a prognosis of retention, as well as the presence of fixed prostheses such as bridges or implants replacing missing teeth. Residual roots indicated for extraction and fully impacted teeth were excluded from the count of remaining teeth. Posterior tooth loss was categorized into two groups: less than 3 teeth missing (<3 teeth) and 3 or more teeth missing (≥3 teeth).

### 2.2. Condylar Changes

**Figure 1:**
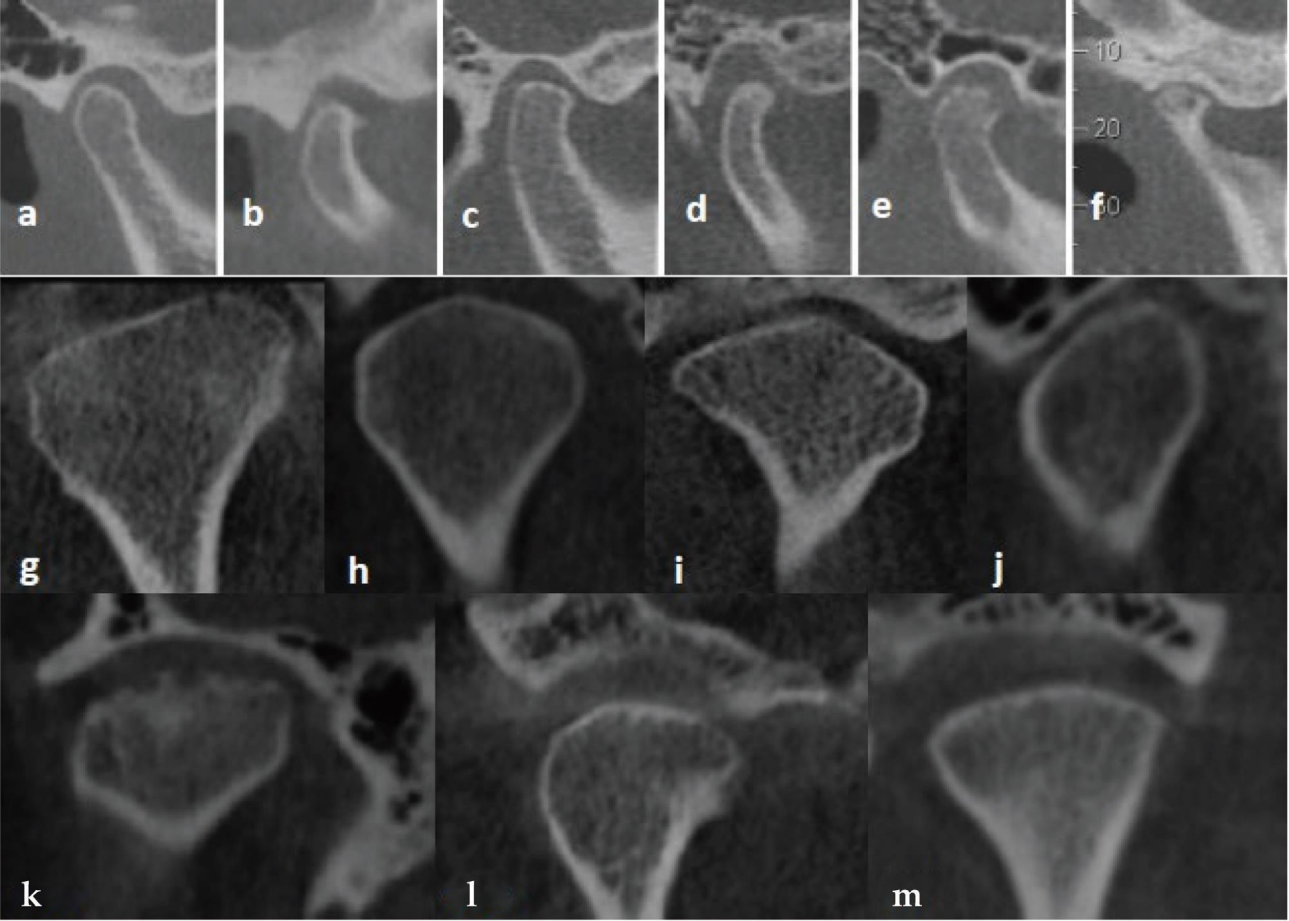
The classification of condylar change, condyle morphology, and cortical mandibular condyle (CMC): condylar change: (a) without condylar change; (b) osteophyte; (c) flattening; (d) sclerosis; (e) erosion; and (f) pseudocyst. Condyle morphology: (g) flat; (h) convex; (i) angled; and (j) round. Cortical mandibular condyle: (k) CMC 0; (l) CMC 1; and (m) CMC 2.

The mandibular condyle was classified into two categories, those without bone changes and those showing degenerative bone changes, such as osteophytes (marginal bony projections), flattening (loss of convexity or concavity in the joint outline), sclerosis (increased cortical and trabecular bone density in the condyle), erosion (areas of cortical and/or subcortical bone damage), and pseudocysts (round radiolucent areas located beneath the cortical bone or within the trabecular bone) (Figure 1) [7, 9, 10].

### 2.3. Condyle Morphology

Condyle morphology in the coronal section was classified according to Yale's classification [11], including: (a) flat, (b) convex, (c) angled, and (d) round (Figure 1).

### 2.4. CMC

Cortical bone on the condylar process was classified into three categories: CMC 0 = no cortical bone on the articular surface of the mandibular condyle, CMC 1 = partial cortical bone coverage, and CMC 2 = complete cortical coverage of the articular surface of the mandibular condyle (Figure 1; CMC 0, CMC 1, and CMC 2) [6].

### 2.5. Condyle Dimensions

**Figure 2:**
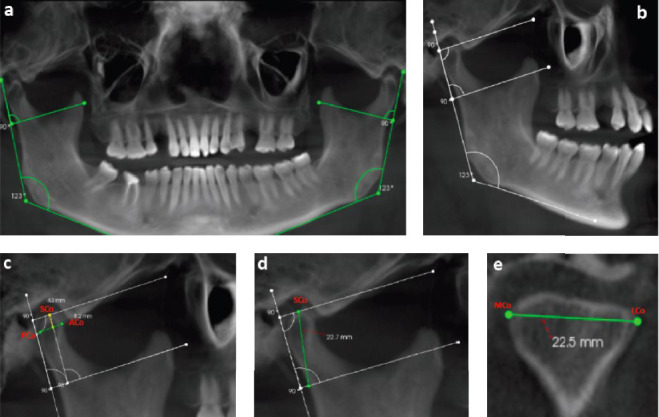
Condyle dimension: (a) panoramic reconstruction; (b) sagittal slicing; (c) condylar length; (d) condylar height; and (e) condylar width.

The area, perimeter, length, height, and width of the mandibular condyle were measured using image analysis software. Panoramic reconstructions and sagittal sections were used to measure the condylar length, height, and width from CBCT images (Figure 2) [4, 5]. Condyle length: In the sagittal CBCT section, the landmarks SCo, ACo, and PCo were used to measure condylar length. The point at the condyle's highest position is called the SCo. A point on the condyle's anterior surface 4 mm below the SCo is called the ACo, and a point on the posterior surface 4 mm below the SCo is called the PCo. The condylar length was determined by measuring a line drawn from ACo to PCo (Figure 2c). Condyle height: In the sagittal CBCT section, the lowest point of the sigmoid notch was marked with a tangent line, which was used to assess condylar height. The horizontal reference line and this tangent line were parallel. A line was drawn linking this crossing point with the SCo when it was determined where the tangent line and the posterior border of the ramus intersected. Condylar height is the measurement of this line (Figure 2d). Condyle width: MCo and LCo were the designated landmarks used to measure condylar width. The mandible's lateral pole is represented by LCo, and its medial pole is denoted the MCo. Maximum mandibular dimensions were displayed in the coronal CBCT portion, where these landmarks were specified. Condylar width in the coronal plane was obtained by measuring the line that was created to link MCo and LCo (Figure 2e).

### 2.6. Statistical Analysis

To describe the measured sample, the data were stratified by sex. When appropriate, Fisher's exact test and Pearson's chi-square test were used to analyze categorical data. First, normality tests were run for numerical data compared to categorical variables. An independent *t*-test was performed if the data were normally distributed; if not, the Mann–Whitney *U* test was used. To examine the causal relationships between variables and condylar changes, condylar changes were categorized into two groups, no change (normal classification) and changes (classified as osteophyte, flattening, erosion, sclerosis, or pseudocyst). Some categorical classifications with a value of 0, such as pseudocyst in condylar changes, were combined. Similarly, CMC classifications of 0 and 1 were merged for analysis. Finally, to assess predictive models for risk factors associated with condylar changes, logistic regression was employed using a stepwise forward conditional method. Condylar changes were used as the dependent variable (0 = no change/normal; 1 = osteophyte, flattening, erosion, sclerosis). Significant independent variables included sex (0 = woman; 1 = man) and CMC (0 = CMC classification 2; 1 = CMC classifications 0 and 1). This study employed logistic regression to explore significant associations between variables and the presence of condylar changes. The regression model was not intended as a diagnostic prediction tool, and thus, diagnostic accuracy metrics such as AUC, sensitivity, and specificity were not calculated. Instead, odds ratios (ORs) with 95% confidence intervals and significance levels (*p*-values) were reported to interpret the strength and direction of the associations. Statistical analyses were conducted using IBM SPSS version 22 for Windows (IBM Corporation, New York, USA).

## 3. Results

The kappa agreement results for intra and interobserver agreement for the variable “condylar changes” were 0.871 and 0.805, respectively, while the ICC results for “condylar length” were 0.969 and 0.913, respectively. The results of both intra and interobserver agreement tests indicated “almost perfect agreement.”

The study population included both men and women, with a notable difference in age between the two groups (Table 1). The median age for men was 51.53 years (range 32–70 years), while for women, it was 37.87 (30–74) years, and this difference was statistically significant (*p* < 0.001), as assessed by the Mann–Whitney *U* test. Condylar height was significantly greater in men (21.9 ± 3 mm) compared to women (20 ± 2.3 mm), with a *p*-value of 0.01, according to the independent samples test. Condylar width was also measured, with men averaging 19.73 ± 2.5 mm and women averaging 18.73 ± 2.7 mm, although this difference did not reach statistical significance. No statistically significant differences between the sexes were found in condylar length, width, CMC classifications, condylar shape, or condylar changes.

Table 2 presents a comparative analysis of several demographic, dental, and anatomical factors between individuals with and without condylar changes. A significant association was observed between sex and condylar changes, with women exhibiting a higher likelihood of condylar alterations than men (*p*=0.039). The mean condylar height was significantly lower in individuals with condylar changes (20.49 ± 2.5 mm) compared to those without (22.34 ± 3.2 mm), indicating a possible link between reduced condylar height and the presence of osteophytes, flattening, sclerosis, or erosion (*p*=0.017). The study found a significant relationship between CMC classification and condylar changes, with a higher prevalence of changes in individuals classified as CMC class 0 or 1 (*p*=0.021). Other variables, including age, number of remaining teeth, posterior tooth loss, condylar length, and condylar width, did not show statistically significant associations with condylar changes. The data suggest that anatomical factors, specifically sex, condylar height, and CMC classification, may be influential in the occurrence of condylar alterations.

The logistic regression analysis aimed to identify the significant predictors of condylar changes, including osteophyte formation, flattening, sclerosis, and erosion (Table 3). In step 1a, the CMC classification emerged as a significant predictor of condylar changes. Specifically, individuals classified as CMC 0 or 1 were significantly more likely to exhibit condylar changes than those in CMC class 2. The OR of 4.46 indicates that individuals with CMC 0 or 1 are over four times more likely to develop condylar changes than those with CMC 2 (*p*=0.017). However, by including sex as a variable in step 2b, the model showed that men were significantly less likely than women to experience condylar changes. The OR of 0.18 suggests that men are 82% less likely than women to exhibit condylar changes (*p*=0.017). Additionally, including sex in the model further strengthened the association between CMC classification and condylar changes. The OR for CMC 0 or 1 increased to 7.5, indicating that individuals in these classes are 7.5 times more likely to develop condylar changes than those in CMC class 2 (*p*=0.007).

## 4. Discussion

The present study investigated the relationship between various demographic, anatomical, and dental factors and condylar changes, including osteophyte formation, flattening, sclerosis, and erosion. In Table 1, the significant age difference between male and female subjects leaves unresolved potential age-related differences in the manifestation of TMJ disorders. Given that the women were generally younger, age could be a confounding factor in assessing sex differences in condylar changes. The finding that men had significantly greater condylar height than women could reflect inherent anatomical differences between the sexes, which may influence the development of TMJ disorders. This aligns with previous research indicating that condylar dimensions can vary significantly based on sex and may impact the mechanical functioning of the TMJ [12].

In Table 2, our findings demonstrate a significant association between sex and condylar changes, with women likelier than men to exhibit these alterations. This result aligns with the existing literature, which consistently reports a higher prevalence of TMJ disorders in women. The underlying reasons for this sex difference may involve hormonal influences, particularly estrogen, which has been implicated in modulating joint tissue responses and inflammation [13, 14]. The lower odds of condylar changes in men, as indicated by an OR of 0.18, suggest that sex-specific factors play a crucial role in the development of TMJ disorders (Table 3). These findings emphasize the need for sex-specific approaches in both the diagnosis and management of TMJ conditions.

The study also identified CMC classification as a significant anatomical predictor of condylar changes. Individuals with CMC classifications of 0 or 1 were found to be at a significantly higher risk for condylar alterations than those in CMC class 2. Specifically, the odds of developing condylar changes were over four times higher in individuals with CMC 0 or 1 in the initial model and increased to 7.5 times higher when sex was included in the regression model. This suggests that the integrity of the mandibular cortex may be critical in maintaining condylar health, potentially due to its role in distributing mechanical loads and resisting degenerative processes [15, 16].

This combined analysis is novel and clinically relevant, as it recognizes that bone integrity (CMC classification) does not operate in isolation but may be modulated by systemic biological factors such as sex hormones. Prior studies have suggested that estrogen may influence cartilage and subchondral bone response to loading, thereby increasing vulnerability in females when cortical support is compromised [17]. Hence, the interaction between sex and cortication reflects a plausible and underexplored biomechanical-pathophysiological model for condylar degeneration. The interaction between sex and CMC classification further elucidates the pathophysiological mechanisms underlying TMJ disorders. The increased OR for CMC classification when sex is accounted for suggests that these two factors may act synergistically to influence condylar outcomes. Women with compromised mandibular cortex integrity (CMC 0 or 1) appear to be particularly vulnerable to condylar changes, highlighting the importance of considering multiple risk factors in clinical assessments.

The significant associations found between condylar changes, sex, and CMC classification have important clinical implications. These findings suggest that routine assessment of CMC classification and consideration of sex differences should be integrated into the evaluation process for patients at risk of TMJ disorders. Early identification of individuals at higher risk could lead to more targeted interventions, potentially mitigating the progression of condylar changes and improving patient outcomes. Furthermore, these results underscore the need for personalized treatment approaches in TMJ disorder management. For instance, women and individuals with lower CMC classifications might benefit from more frequent monitoring and preventive strategies, such as occlusal adjustments or splint therapy, to reduce the likelihood of condylar deterioration [18].

This study has several limitations that should be acknowledged. First, due to its cross-sectional design, causal inferences cannot be made. Second, although age was considered in the analysis, other potentially relevant variables, such as parafunctional habits (e.g., bruxism or clenching), were not available in the patient data and therefore could not be adjusted for. Third, the modest sample size may limit the generalizability of our findings. Future studies with larger, multicenter samples and longitudinal follow-up are recommended to confirm and extend these results.

## 5. Conclusion

This study's findings confirmed that both sex and CMC classification are significant predictors of condylar changes in TMJ disorders, which suggests that assessing these factors can aid in the early diagnosis and personalized treatment of TMJ disorders, potentially improving patient outcomes.

## Figures and Tables

**Table 1:** Description of the study subjects.

Variables	Men (%)		Women (%)		*p*-Value
Age^b^ (years)	51.53 (32–70)		37.87 (30–74)		0.000 (Mann–Whitney *U* test)
Number of remaining teeth^a^	23.15 ± 8.8		27.58 ± 3		NS
Posterior tooth loss ≥3 teeth	24 (37.5)		22 (34.4)		NS
Posterior tooth loss <3 teeth	9 (14.1)		9 (14.1)		—
Condylar length^a^ (mm)	7.93 ± 1.1		7.7 ± 1.2		NS
Condylar height^a^ (mm)	21.9 ± 3		20 ± 2.3		0.01 (independent samples test)
Condylar width^a^ (mm)	19.73 ± 2.5		18.73 ± 2.7		NS
Cortical mandibular cortex (CMC)					
CMC class 0	0		3 (4.7)		—
CMC class 1	27 (42.2)		19 (29.7)		NS
CMC class 2	6 (9.4)		9 (14.1)		—
Condyle shape	Right	Left	Right	Left	
Flat	9 (14.1)	10 (15.6)	7 (10.9)	8 (12.5)	
Convex	19 (29.7)	16 (25)	15 (23.4)	15 (23.4)	NS
Angled	2 (3.1)	3 (4.7)	3 (4.7)	4 (6.3)	—
Round	3 (4.7)	4 (6.3)	6 (9.4)	4 (6.3)	—
Condylar change	Right	Left	Right	Left	NS
No change	19 (29.7)	22 (34.4)	10 (15.6)	14 (21.9)	—
Osteofit	6 (9.4)	5 (7.8)	9 (14.1)	7 (10.9)	—
Flattening	1 (1.60	2 (3.1)	4 (6.3)	2 (3.1)	—
Sclerosis	3 (4.7)	2 (3.1)	3 (4.7)	3 (4.7)	—
Erosion	4 (6.3)	2 (3.1)	5 (7.8)	5 (7.8)	—
Pseudocyst	0	0	0	0	—

Abbreviation: NS, not significant.

^a^Mean ± standard deviation.

^b^Median (minimum–maximum).

**Table 2:** Relationship of condylar changes with all variables.

Variables	No change	Osteofit, flattening, sclerosis, erosion	*p*-Value
Age (years)^b^	50.5 (30–69)	42 (30–74)	NS
Sex: men	13 (20.3)	20 (31.3)	0.039 (Pearson's chi-square)
Sex: women	5 (7.8)	26 (40.6)	—
Number of remaining teeth^b^	27.5 (5–32)	27.5 (0–32)	NS
Posterior tooth loss ≥3 teeth	13 (20.3)	33 (51.6)	NS
Posterior tooth loss <3 teeth	5 (7.8)	13 (20.3)	—
Condylar length (mm)^b^	8.3 (5.5–9.3)	7.9 (4.5–10)	NS
Condylar height (mm)^a^	22.34 ± 3.2	20.49 ± 2.5	0.017 (independent samples test)
Condylar width (mm)^a^	19.78 ± 1.8	19.04 ± 2.8	NS
CMC class 2	8	7	0.021 (Fisher's exact test)
CMC class 0 and 1	10 (15.6)	39	—
Condyle shape right: flat	2 (3.1)	14 (21.9)	NS
Condyle shape right: convex	10 (15.6)	24 (37.5)	—
Condyle shape right: angled	3 (4.7)	2 (3.1)	—
Condyle shape right: round	3 (4.7)	6 (9.4)	—
Condyle shape left: flat	3 (4.7)	15 (23.4)	NS
Condyle shape left: convex	11 (17.2)	20 (31.3)	—
Condyle shape left: angled	2 (3.1)	5 (7.8)	—
Condyle shape left: round	2 (3.1)	6 (9.4)	—

^a^Mean ± standard deviation.

^b^Median (minimum–maximum).

**Table 3:** Logistic regression analysis using condylar change as the dependent variable.

Condylar change (0: no change; 1: osteofit, flattening, sclerosis, and erosion)	*B*	Standard error	Wald	Odds ratio	*p*-Value
Step 1: CMC (0 = class 2; 1 = classes 0 and 1)	1.5	0.63	5.68	4.46	0.017
Step 2: Sex (0 = women; 1 = men)	−1.73	0.73	5.67	0.18	0.017
Step 2: CMC (0 = class 2; 1 = classes 0 and 1)	2.01	0.75	7.2	7.5	0.007
Constant	0.512	0.61	0.7	1.67	0.403

## Data Availability

The data that support the findings of this study are available upon request from the corresponding author. The data are not publicly available due to privacy or ethical restrictions.
